# Cost-effectiveness of breast cancer screening using mammography in Vietnamese women

**DOI:** 10.1371/journal.pone.0194996

**Published:** 2018-03-26

**Authors:** Chi Phuong Nguyen, Eddy M. M. Adang

**Affiliations:** 1 Department of Pharmaceutical Administration and Economics, Hanoi University of Pharmacy, Hanoi, Vietnam; 2 Department of Health Evidence, Radboud University Nijmegen Medical Centre, Nijmegen, the Netherlands; University of Zurich, SWITZERLAND

## Abstract

**Background:**

The incidence rate of breast cancer is increasing and has become the most common cancer in Vietnamese women while the survival rate is lower than that of developed countries. Early detection to improve breast cancer survival as well as reducing risk factors remains the cornerstone of breast cancer control according to the World Health Organization (WHO). This study aims to evaluate the costs and outcomes of introducing a mammography screening program for Vietnamese women aged 45–64 years, compared to the current situation of no screening.

**Methods:**

Decision analytical modeling using Markov chain analysis was used to estimate costs and health outcomes over a lifetime horizon. Model inputs were derived from published literature and the results were reported as incremental cost-effectiveness ratios (ICERs) and/or incremental net monetary benefits (INMBs). One-way sensitivity analyses and probabilistic sensitivity analyses were performed to assess parameter uncertainty.

**Results:**

The ICER per life year gained of the first round of mammography screening was US$3647.06 and US$4405.44 for women aged 50–54 years and 55–59 years, respectively. In probabilistic sensitivity analyses, mammography screening in the 50–54 age group and the 55–59 age group were cost-effective in 100% of cases at a threshold of three times the Vietnamese Gross Domestic Product (GDP) i.e., US$6332.70. However, less than 50% of the cases in the 60–64 age group and 0% of the cases in the 45–49 age group were cost effective at the WHO threshold. The ICERs were sensitive to the discount rate, mammography sensitivity, and transition probability from remission to distant recurrence in stage II for all age groups.

**Conclusion:**

From the healthcare payer viewpoint, offering the first round of mammography screening to Vietnamese women aged 50–59 years should be considered, with the given threshold of three times the Vietnamese GDP per capita.

## Introduction

Globally, breast cancer is the most prevalent cancer in women. In terms of mortality, breast cancer becomes the leading cause of cancer related death in women in developing countries and the second leading cause in developed countries [1–3]. Approximately 1.67 million new breast cancer cases were diagnosed (25% of all cancers) and 522,000 deaths from breast cancer occurred in 2012 all over the world [4]. Although breast cancer is thought to be a common female disease in high-income countries, nearly 50% of breast cancer cases and 58% of deaths due to breast cancer occur in low- and middle-income countries where the majority of women were diagnosed at the late stages [5]. Breast cancer is also the most common cancer among women in Vietnam. The incidence rate rose from 13.8 per 100,000 Vietnamese women (in 2000) to 28.1 per 100,000 Vietnamese women (in 2010). According to Duc NB, 12,533 women with breast cancer were diagnosed in 2010 [6]. Potentially explanatory factors for the rising rate of breast cancer in Vietnamese women were: the fall in fertility rate, the increase in overweight women, and the improved healthcare system (i.e., the increased availability of diagnostic tests) [7]. The peak age group of breast cancer was 50–54 years in Vietnamese women. Breast cancer is the third leading cause of death from cancer; only liver cancer and lung cancer account for more cancer deaths in Vietnamese women [8].

Early detection can improve breast cancer outcomes and the survival of breast cancer patients. The highest proportion of breast cancer patients in low and middle-income countries, including Vietnam, are diagnosed at the late stages which makes the treatment more difficult and costly [7]. Therefore, breast cancer screening in the general population could potentially reduce both the health burden and the economic burden of breast cancer. Although there are currently some breast cancer screening approaches, only mammography screening has been proven to reduce breast cancer mortality by early detection in population-based programs [2].

National mammography screening programs have been widely implemented including in Europe, North America, and Australia and the cost-effectiveness of these schemes has been published [9–12]. An estimated cost per life year gained of US$27,000 was found in the US [13] and US$19,919 in Australia [14]. In Asian countries, the cost per life year gained had a large range from US$19,257 (India) [15] and US$29,964 (South Korea) [16] up to US$64,400 (China) [17]. However, the guidelines of optimal screening policies (start age, end age, and interval screening) differ between countries. The American Cancer Society recommends that annual mammography screening for women begins at age 45 [18]. The Department of Health of the Australian Government recommends women aged between 50 and 74 years for biennial mammography screening. Factors such as age, breast tissue density, and screening interval, affect the sensitivity and specificity of mammography screening. Some Asian countries, such as South Korea, Japan, and Singapore, have initiated population-based screening programs. In South Korea, women are being screened with mammography every two years, starting at age 40 [19]. The Ministry of Health of Singapore recommends that biennial mammography screening should be implemented for women between 50–69 years [20]. According to Chisato Hamashima et al., Japanese women between 40–74 years of age should be screened by mammography without clinical breast examination for population-based screening [21].

National mammography screening is presently not performed in Vietnam; there is only private screening (patients pay the screening fees) which hampers nationwide screening due to the financial barrier. The rate of uptake in private screening and information on the epidemiology of screened women are not available in Vietnam. It is not possible to perform the national mammography screening program for all Vietnamese women since the age-adjusted incidence rate is lower than that in other countries and the budget of the healthcare system is very limited. Therefore, the aim of this study is to estimate: (a) the cost-effectiveness of mammography screening among Vietnamese women; (b) the appropriate age category for the first round of mammography screening among Vietnamese women.

## Methods

### Model structure and assumptions

**Fig 1:**
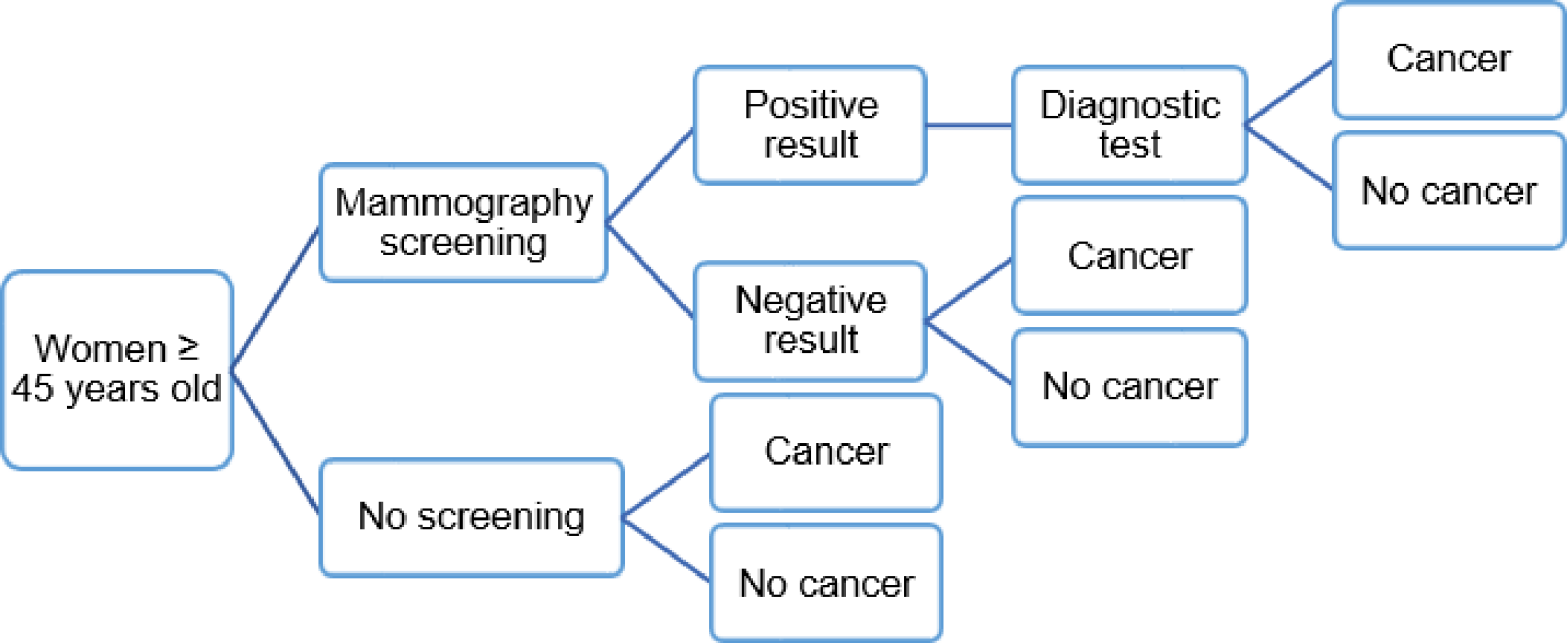
Decision tree model.

**Fig 2:**
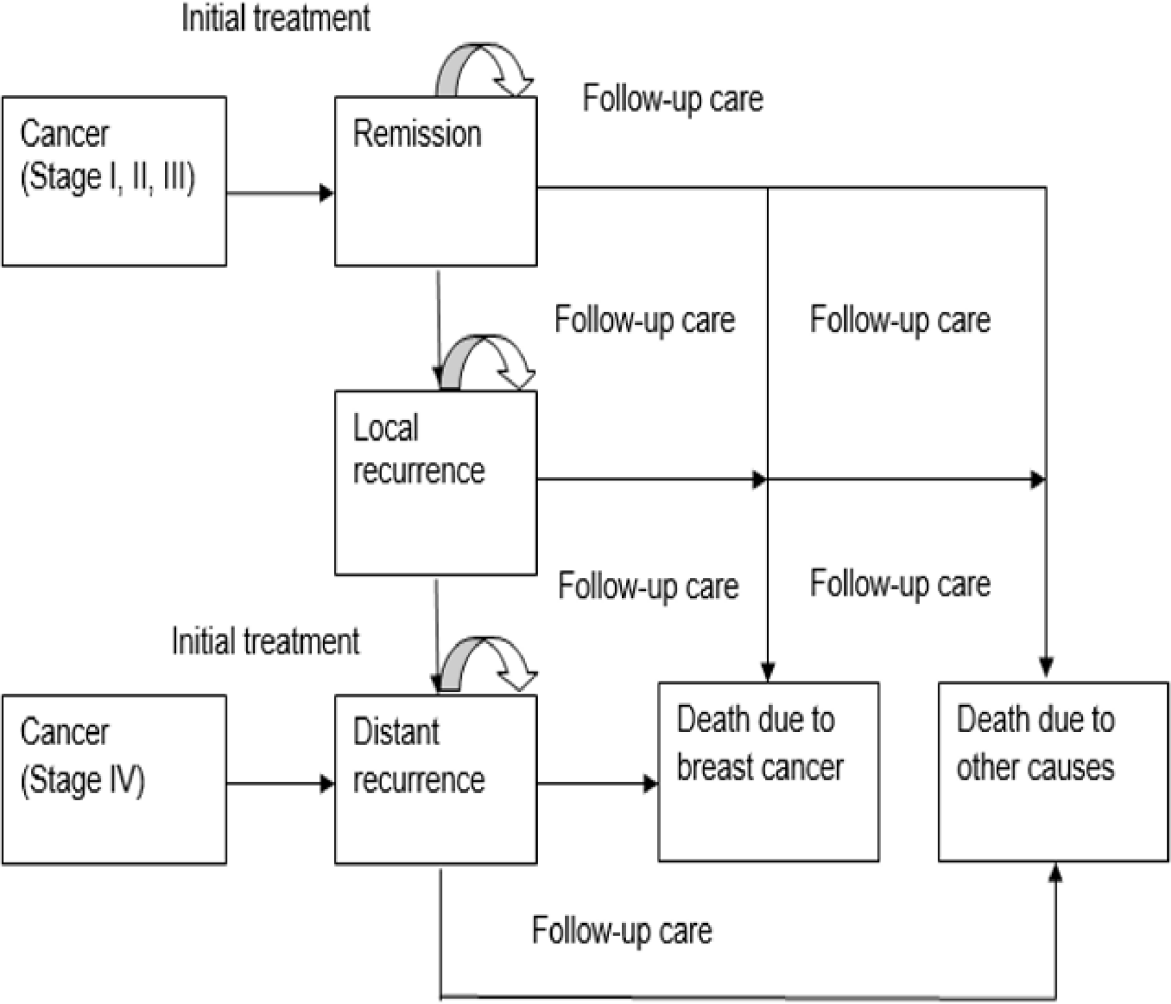
Health states and transitions.

The screening strategy under evaluation is that women over 45 years of age are being screened by mammography. Four age groups were considered: 45–49, 50–54, 55–59, and 60–64 years. It was assumed that 100% of the target population would participate in the screening program. The impact of the participation rate on the cost-effectiveness of mammography screening was evaluated ranging from a 100% participation rate to a 23.6% participation rate which was derived from the national breast cancer screening program in Korea [22]. The costs and outcomes of mammography screening were compared with: (1) no screening (base case); (2) a combination of 5% private screening and 95% no screening; (3) a combination of 10% private screening and 90% no screening. A decision analytical modeling approach was used to detect patients with breast cancer (Fig 1). Women with an abnormal mammogram were taken for an extra laboratory test to diagnose breast cancer and were determined by the stage of cancer: stage I, stage II, stage III, stage IV or metastatic stage. We assumed that all breast cancer patients underwent prompt and adequate treatment. Women with a determined stage of cancer entered a Markov model linked to the decision tree model (Fig 2).

Markov chain analysis was used to calculate costs and life years gained due to early detection of breast cancer. Entering the Markov model depends on the stage patients are being diagnosed. The five states in the Markov model are: remission (after treatment), local recurrence, distant recurrence, death due to breast cancer, and death due to other causes. In each one-year cycle, women can enter the remission state and remain there or make a transition to local recurrence, distant recurrence, death due to breast cancer, or death due to other causes. Women in the local recurrence state can remain in this state or make a transition to distant recurrence, death due to breast cancer or death due to other causes. Finally, women in the distant recurrence (including stage IV) can remain at the same state or make a transition to death due to breast cancer or death due to other causes [23]. The Markov model was run with a lifelong time horizon.

### Model input parameters

#### Epidemiological parameters

Key parameters are summarized in Table 1. All-cause mortality probabilities by age were obtained from data published by the General Statistics Office of Vietnam [24]. We used age-specific breast cancer incidence figures from online data of the International Agency for Research on Cancer [8]. Breast cancer stage distributions in the non-screened group were derived from a Vietnamese published article [25] while stage distributions in the screened group were obtained from the Chinese article of Wong et al. [17] as these parameter estimates were not available in Vietnam. Ductal carcinoma in situ (DCIS) was excluded from the model since epidemiological parameters related to DCIS were not recorded in Vietnam. Due to the lack of data, it was assumed that stage distributions in the screened group and the non-screened group were the same for each age group. We applied transition probabilities between health states in the Markov model from a Canadian study [23]. It was also assumed that transition probabilities between health states for the 60–64 age group were the same as that for the 50–59 age group. The survival probability of patients with stage IV breast cancer was estimated from a Vietnamese study [26] and was used to recalculate transition probabilities between health states in the Markov chain for scenario analyses (S1 Table).

**Table 1:** Complete model input.

Parameter	Value	Distribution	Reference
**Epidemiology**			
***All-cause mortality*: *age distribution***		Invariant	Vietnam [24]
*45–49*	0.0037		
*50–54*	0.0058		
*55–59*	0.0094		
*60–64*	0.0159		
*65–69*	0.0271		
*70–74*	0.0452		
*75–79*	0.0680		
*80+*	0.1129		
***Breast cancer incidence*: *age group***		Beta^a^	Vietnam [8]
*45–49*	0.000689		
*50–54*	0.000829		
*55–59*	0.000818		
*60–64*	0.000685		
***Breast cancer stage distribution*: *screened***		Dirichlet^b^	China [17]
*Stage I*	0.521		
*Stage II*	0.382		
*Stage III*	0.057		
*Stage IV*	0.041		
***Breast cancer stage distribution*: *non-screened***		Dirichlet^b^	Vietnam [25]
*Stage I*	0.1118		
*Stage II*	0.6118		
*Stage III*	0.1941		
*Stage IV*	0.0823		
***Stage progression transition probabilities***		Invariant	Canada [23]
Age group 45–49			
*Stage I*			
*Remission to Local recurrence*	0.01		
*Remission to Distant recurrence*	0.000016		
*Local recurrence to Distant recurrence*	0.062		
*Local recurrence to breast cancer death*	0.013		
*Distant recurrence to breast cancer death*	0.555		
*Stage II&III*			
*Remission to Local recurrence*	0.018		
*Remission to Distant recurrence*	0.024		
*Local recurrence to Distant recurrence*	0.165		
*Distant recurrence to breast cancer death*	0.386		
*Stage IV*			
*Distant recurrence to breast cancer death*	0.386		
Age group 50–59			
*Stage I*			
*Remission to Local recurrence*	0.009		
*Remission to Distant recurrence*	0.000025		
*Local recurrence to Distant recurrence*	0.052		
*Distant recurrence to breast cancer death*	0.137		
*Stage II&III*			
*Remission to Local recurrence*	0.016		
*Remission to Distant recurrence*	0.105		
*Local recurrence to Distant recurrence*	0.13		
*Distant recurrence to breast cancer death*	0.423		
*Stage IV*			
*Distant recurrence to breast cancer death*	0.423		
Survival probability of patients in stage IV	0.50	Invariant	Vietnam [26]
**Mammography test**			
***Sensitivity*: *age group***		Beta^c^	Japan [27]
*45–49*	0.714		
*50–59*	0.858		
*60–64*	0.872		
***Specificity*: *age group***		Beta^c^	
*45–49*	0.886		
*50–59*	0.907		
*60–64*	0.931		
**Cost (US$)**			
***Mammography test***	8.15	Invariant	Vietnam [28]
***Meeting with doctor***	1.75	Invariant	
***Cost of further testing***		Invariant	
*Biopsy*	6.45		
*Cytological test*	4.66		
Treatment costs*			Vietnam [26]
Initial treatment cost		Gamma^d^	
*Stage I*	160.00		
*Stage II*	458.48		
*Stage III*	850.45		
*Stage IV*	668.70		
Follow-up cost	88.57	Gamma^d^	

^a^ *Parameters of the Beta distribution were derived from the number of cancer cases relative to the population by age groups*.

^*b*^ *Parameter means of the distribution were equivalent to the sample size in each stage*. *Dirichlet was [1241;911;135;97] in the screened group and Dirichlet was [106;580;184;78] in the non-screened groups*.

^*c*^ *Parameters of the Beta distribution were derived from the number of cases having a positive result*, *a negative result*, *cancer*, *and no cancer*.

* Costs were adjusted by the Vietnamese Consumer Price Index to the year 2016.

^*d*^ *The standard deviations for the initial treatment costs of stage I*, *II III and IV were US$190*.*76*, *US$546*.*64*, *US$1013*.*98*, *US$960*.*05*, *respectively*. *The standard deviation for costs of follow-up care was US$66*.*47*.

#### Sensitivity and specificity of mammography screening

Due to the lack of data about the accuracy of mammography screening in Vietnamese women, the sensitivity and specificity of mammography screening programs were derived from a Japanese study [27].

#### Costs

The study was conducted from the viewpoint of the healthcare payer. Direct non-medical costs and indirect costs (like productivity losses) were not included in this study. The costs of tests were derived from a list of the Vietnamese Ministry of Health [28] and converted to the US dollar (US$ 1 = VND 22,330). In Vietnam, there are main laboratory tests to diagnose breast cancer, including tumor biopsy, cytological tests, ultrasound, and CA15.3 [26]. In this model, we assumed that among women with an abnormal mammogram, 50% required biopsy and 50% required cytological testing. Treatment costs were obtained from a previously published article of Nguyen Hoang Lan et al. [26]. These costs were adjusted to the year 2016 based on the Vietnamese Consumer Price Index [29] and included the initial treatment costs and follow-up costs. It was assumed that treatment costs were the same for each age group and the follow-up costs were the same for each cycle in the Markov model.

### Outcomes

The model outcomes were: life year gained and total costs. The comparative performance of the strategies was measured by using an Increment Cost Effectiveness Ratio (ICER) and/or an Incremental Net Monetary Benefit (INMB). The ICER is defined as the difference in the total costs between the new screening program and the no screening alternative, divided by the difference in the effectiveness between these two options. The Net Monetary Benefit measures both cost and health outcome in terms of monetary value. Therefore, the INMB is calculated as the difference in effectiveness between both alternatives multiplied by a given willingness-to-pay threshold for a unit of effect gained, and then the difference in cost between both alternatives is subtracted from it [30]. The mammography screening is considered cost-effective if the ICER was less than 3 times the GDP per capita according to the WHO recommendation [31]. Both future health effects and costs were discounted at an annual rate of 3%. All analyses were conducted using the TreeAge Pro 2016 software.

### Uncertainty analyses

To check the robustness of our results, we conducted one-way sensitivity analyses presented through a Tornado diagram. The parameters included in the one-way sensitivity analyses were as follows: discount rate, initial treatment cost, follow-up cost, mammography sensitivity, mammography specificity, and transition probabilities for the health states. Based on the literature, we determined a range of plausible values for the parameters in the model. In cases in which ranges could not be found from the previously published documents, the range of values that was used ±25% of the base-case as a conservative estimate (Table 2).

**Table 2:** Parameters and value ranges used for one-way sensitivity analysis.

**Parameter**	**Low value**	**High value**
Mammography sensitivity	75% of base-case value	125% of base-case value
Mammography specificity	75% of base-case value	125% of base-case value
Transition rate from distant recurrence to cancer death	50% of base-case value	150% of base-case value
Transition rate from local recurrence to distant recurrence	50% of base-case value	150% of base-case value
Initial treatment cost	75% of base-case value	125% of base-case value
Follow-up cost	75% of base-case value	125% of base-case value
Discount rate	0%	6%

A probabilistic sensitivity analysis was performed to examine parameter uncertainty using second order Monte Carlo simulation with 10,000 iterations. In conducting a probabilistic sensitivity analysis, the distribution of each parameter was defined based on literature (Table 1). This analysis used the cost-effectiveness ceiling threshold (the willingness-to-pay threshold for a life year gained) of three times GDP per capita. The Vietnamese GDP per capita in 2015 was US$2,110.90 and the ceiling threshold, therefore, was US$6,332.70 per life year gained [32].

### Ethics consideration

The Ethics Committee approval was not required for this cost-effectiveness study as model parameters in this study were obtained from previously published data.

## Results

Table 3 shows the estimated average total costs and average total life years gained per 100,000 women under different screening strategies. Among the screening policies that were assessed, the first round of mammography screening in the 50–54 age group had the lowest ICER (US$3,647.06 per life year gained) and highest INMB (US$775,674). Mammography screening for women aged 55–59 years was estimated to gain 289 life years per 100,000 women and resulted in an ICER of US$4,405.44 when compared to no screening. According to the INMB decision rule (INMB>0), screening for the 45–49 age group and the 60–64 age group could not be considered cost-effective since INMBs were negative.

**Table 3:** Costs and effects for comparison of screening strategies for 100,000 Vietnamese women.

Strategy	Cost (US$)	Life year gained	ICER (US$) per patient	INMB (US$)
**Women aged 45–49 years**				
No screening	115,603	100,520		
Mammography screening	1,169,942	100,640	8,782.70	-294,117
**Women aged 50–54 years**				
No screening	104,134	100,096		
Mammography screening	1,157,487	100,385	3,647.06	775,674
**Women aged 55–59 years**				
No screening	99,351	99,689		
Mammography screening	1,148,683	99,928	4,405.44	459,055
**Women aged 60–64 years**				
No screening	79,603	98,897		
Mammography screening	1,110,692	99,060	6,335.84	-512

Furthermore, the impact of opportunistic screening and the participation rate on the cost-effectiveness of mammography screening were explored since the rate of uptake in private screening and the participation rate in mammography were not available in Vietnam. The results showed that changing the percentage of individual screening from 0% to 10% had a minor effect on the cost-effectiveness of mammography screening (Table 4). In fact, the INMB turned out to be insensitive for changes in all screening policies: 100% no screening, 95% no screening + 5% individual screening and 90% no screening + 10% individual screening. However, changing the participation rate from 100% to 23.6% had a high impact on the INMBs of all age groups and mammography screening in the 50–54 age group, as well as the 55–59 age group, was considered cost-effective with an INMB of US$183,059 and US$108,337, respectively.

**Table 4:** INMBs for comparison of changing the percentage of individual screening and the participation rate.

Group	INMB (US$) of base case	INMB (US$) of Screening vs.		INMB (US$) of 23.6% screening vs. no screening
		95% no screening+ 5% individual screening	90% no screening+ 10% individual screening	
45–49 years	-294,117	-279,465	-264,815	-69,412
50–54 years	775,674	736,811	697,948	183,059
55–59 years	459,055	436,289	413,244	108,337
60–64 years	-512	-486	-460	-121

Due to the lack of Vietnamese data concerning transition probabilities between health states in the Markov model, parameters from a Canadian study were used. The average total cost, average total life years gained, the ICER and the INMB were analyzed by substitution of the survival rate of stage IV in Vietnam for that of the parameters in Canada and recalculation of transition probability parameters (Table 5). All ICERs of screening policies were lower than their counterparts in the base case. However, screening for the 45–49 age group was not considered to be cost-effective regarding the threshold of US$6332.70 since the ICER in this group was US$7,680.08. With these adjusted transition probability parameters, the ICER of mammography screening for women aged 60–64 reduced from US$6,335.84 to US$5,799.90 and was considered cost-effective.

**Table 5:** Costs and effects of screening policies when using adjusted transition probability parameters.

Strategy	Cost (US$)	Life year gained	ICER (US$)	INMB (US$)
**Women aged 45–49 years**				
No screening	107,665	100,430		
Mammography screening	1,163,548	100,568	7,680.08	-185,243
**Women aged 50–54 years**				
No screening	98,241	100,030		
Mammography screening	1,153,423	100,339	3,409.65	904,592
**Women aged 55–59 years**				
No screening	94,066	99,630		
Mammography screening	1,145,132	99,887	4,077.55	581,306
**Women aged 60–64 years**				
No screening	75,724	98,853		
Mammography screening	1,108,165	99,031	5,799.90	94,843

### Sensitivity analyses

**Fig 3:**
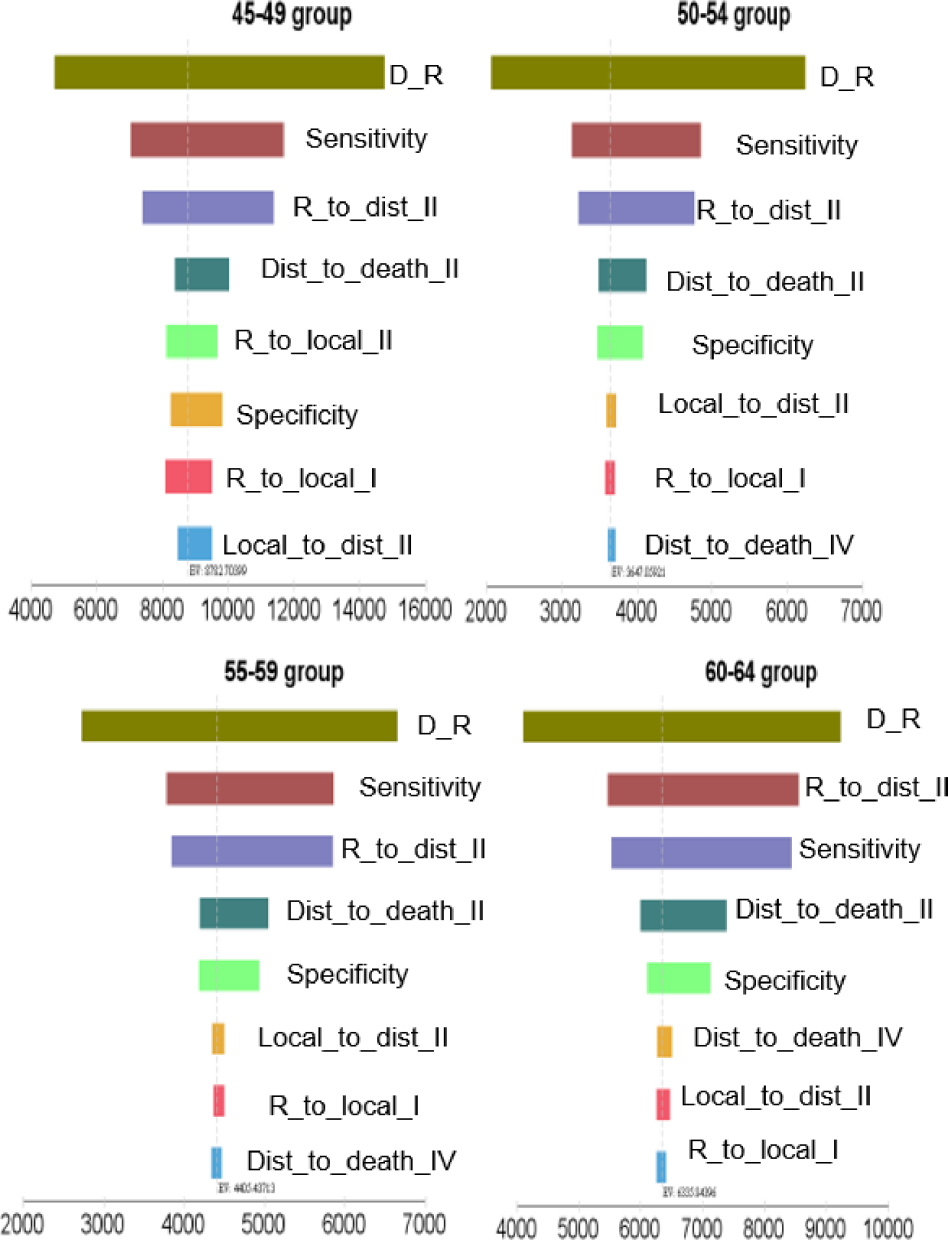
Impact of the parameters on the ICERs. D_R means discount rate; Dist_to_death_II: transition probability from distant recurrence to death in stage II; Dist_to_death_IV: transition probability from distant recurrence to death in stage IV; Local_to_dist_II: transition probability from local recurrence to distant recurrence in stage IV; R_to_dist_II: transition probability from remission to distant recurrence in stage II; R_to_local_I: transition probability from remission to local recurrence in stage I; R_to_local_II: transition probability from remission to local recurrence in stage II; Sensitivity: sensitivity of mammography; Specificity: specificity of mammography.

To test the impact of certain parameters on the ICERs, one-way sensitivity analyses were conducted for four screening policies. The eight parameters had an impact on the ICERs presented in Fig 3. The ICERs were most sensitive to the ‘*discount rate’* in the four screening policies. *‘Mammography sensitivity’* had the second-highest impact on the ICERs in the three age groups: 45–49, 50–54, and 55–59 while *‘transition probability from remission to distant recurrence in stage II’* ranked the second among the parameters having a high effect on the ICER in the 60–64 age group.

**Fig 4:**
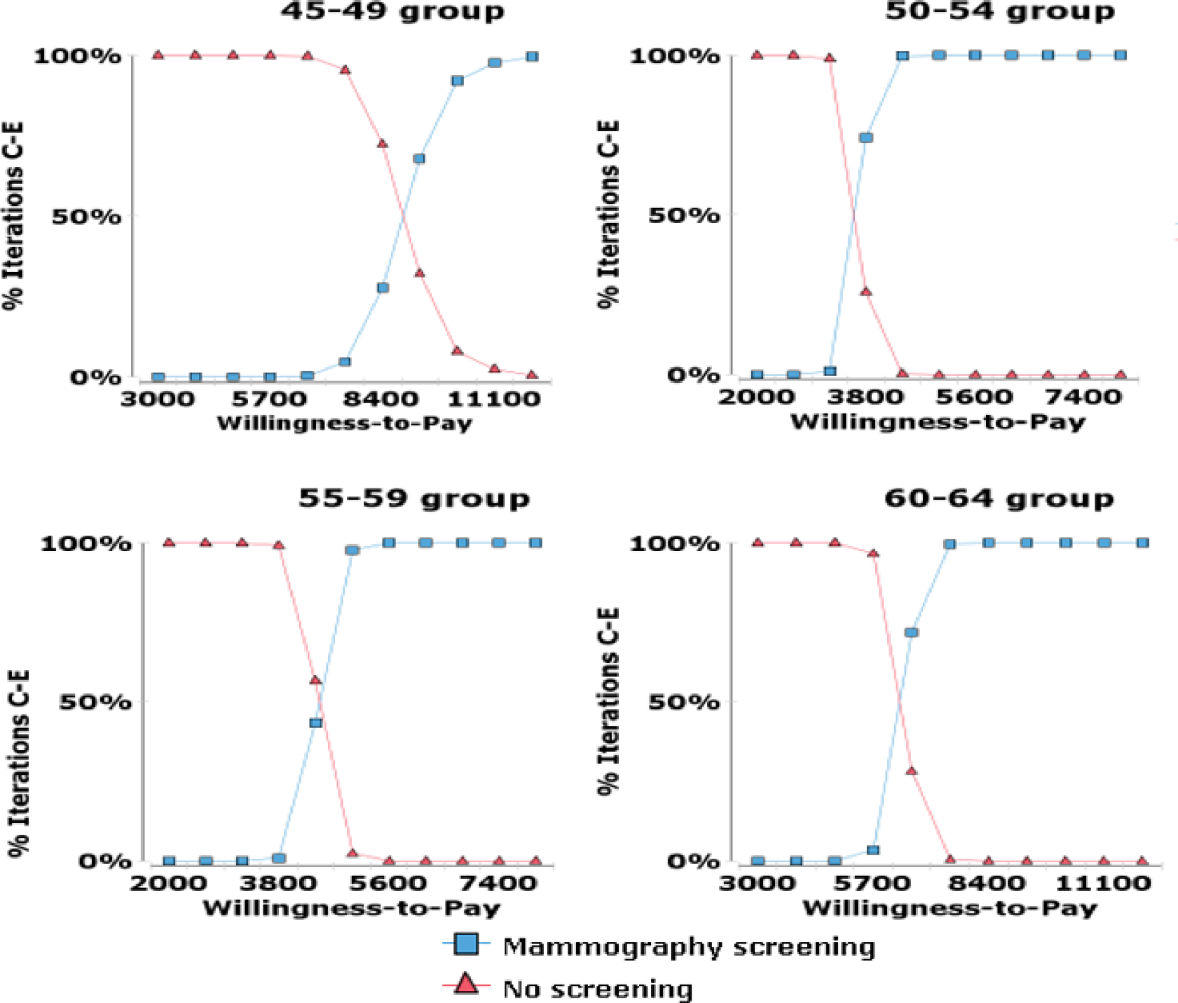
Cost-effectiveness acceptability curves for screening policies.

We performed a probabilistic sensitivity analysis to test the robustness of the model. Fig 4 shows cost-effectiveness acceptability curves of four strategies based on different ceiling thresholds (Fig 4). Both screening for the 50–54 age group and the 55–59 age group would be cost-effective compared to no screening at the ceiling threshold of US$6,332.70 per life year gained. Compared to no screening for women aged 60–64, screening had a lower probability of being cost-effective at the ceiling threshold since the screening program turned out to be cost-effective in only 46.32% of cases. Fig 4 illustrates that 0% of cases were cost-effective at the threshold if women aged 45–49 were screened.

## Discussion

Low-cost screening approaches, such as clinical breast examination, could be implemented in limited resource settings as the WHO recommends. Presently, there are some studies regarding clinical breast examination as a method of breast cancer screening in Vietnam [33, 34]. However, these publications were cross-sectional studies with a small number of subjects and did not provide the necessary information on parameters such as sensitivity, specificity of clinical breast examination, and stage-specific diagnosis. So far, the only breast cancer screening method that has been proven to be effective by early detection is mammography screening [2]. Accordingly, we analyzed the cost-effectiveness of mammography screening as compared to no screening in Vietnamese women.

Although mammography screening for early detection of breast cancer has been adopted in many Western countries, there are no data related to the effectiveness of mammography screening in Vietnam. As a result, we used parameters of breast cancer which were available in Vietnam and used other parameters from other countries to analyze the cost-effectiveness of mammography screening in Vietnamese women. Our results demonstrated that a single screening in the 50–54 age group, as well as the 55–59 age group, was cost-effective at the given ceiling threshold of US$6,332.7, while screening for women aged 45–49 years and women aged 60–64 years were not considered cost-effective. The reasons for this were that the incidence rate of breast cancer in these groups is lower than that of the other groups [8] and the mammography sensitivity in women in their 40s is relatively lower than for the older age groups [27, 35]. In addition, results from randomized trials showed no significant effect on breast cancer mortality in women between 40–49 years of age that were offered screening [36].

We also performed the cost-effectiveness of mammography screening in the second round for four specific age groups and only screening in women aged 50–54 years was considered cost-effective with an ICER of US$5,090.83 per life year gained (See S2 Table). This implies that increasing the screening frequencies for women aged 45–64 years will increase life years gained, but with decreasing cost-effectiveness of screening. More studies reported that cost per life year gained was reduced as the screening interval increased [23, 37, 38]. Annual and biennial mammography screening for women aged 45–64 years is not cost-effective because the ceiling threshold is quite low in Vietnam. However, we estimated the cost-effectiveness of the second round based on a lot of assumptions due to the lack of data, including similar stage distribution and transition probabilities as for the first round. It is expected that less aggressive cancer is in the subsequent screening in clinical practice. Consequently, long follow-up studies with a sufficient number of subjects should be conducted to estimate effectiveness of mammography screening in Vietnamese women.

We also analyzed the impact of opportunistic screening and the participation rate on the cost-effectiveness of mammography screening because of data scarcity in Vietnam. Our findings showed that changing individual screening from 0% to 10% had a minor effect on the cost-effectiveness of mammography screening while reducing the participation rate to 23.6% had a high impact on the INMBs of all screening policies because of the large effect on total costs. This was also found in the Canadian study [23].

The results of this study demonstrated that the discount rate, mammography sensitivity, and the transition rate in health states had a substantial impact on the ICER in all four groups. An important limitation in our study is the lack of data on mammography sensitivity for age-specific groups of Vietnamese women and we, therefore, had to use data from a Japanese study [27]. Factors such as age, screening interval, breast density, experience of trained professionals, and medical equipment could affect the sensitivity of mammography screening which were assumed to be similar to the Japanese condition.

Another limitation is that we applied transition probabilities between health states from a Canadian study. However, we adjusted these transition probability parameters based on the survival probability of stage IV of Vietnamese breast cancer patients which was lower than that of Canada. The number of deaths from breast cancer as a percentage of incident cases in low-income countries in 2008 was twice as high as that in high-income countries [39, 40]. The reasons for these high mortality rates due to breast cancer are the lack of awareness of breast cancer patients, inadequate facilities for detection and diagnosis, and poor access to high-quality treatment options in low and middle-income countries [40]. Breast cancer patients diagnosed at the late-stages of breast cancer had significantly higher initial treatment costs than that at early-stages, while their survival probability was lower [26]. We found that all screening policies were more cost-effective when using adjusted transition probability parameters. Therefore, early detection of breast cancer through the national screening program would probably result in substantial improvements for the survival rate of patients in low- and middle-income countries whilst the treatment of breast cancer is still limited.

As for Vietnam, there is no information available on the rate of tests used to diagnose after screening. Therefore, only costlier invasive tests were included in the model to not underestimate the total cost associated with screening for breast cancer. We acknowledge that more non-invasive tests like ultrasound can also be part of the post-screening diagnosis pathway. After breast cancer patients complete an initial treatment, they are often required to continue treatment with follow-up care treatment and a range of methods is used in follow-up care depending on characteristics of patients and disease status, such as breast cancer stage, local recurrence and/or distant recurrence [26]. Due to the lack of such data, we assumed that cost for follow-up care was the same for all stage-specific diagnosis and all age groups. However, our one-way sensitivity analyses showed that the ICERs of all groups were insensitive to follow-up cost as well as initial treatment cost. Wong et al. also found that treatment costs had a minor impact on the ICER of breast cancer screening in Chinese women [17].

It has been well-known that the false-positive result is one of the harms of mammography screening [41]. The risk of false-positive results is higher in young women [42]. It leads to additional testing, such as biopsy, and ultrasound test, and is related to greater anxiety about breast cancer or required psychological support [43]. Nevertheless, Maria et al. revealed that women who experienced the false-positive mammogram still considered the false-positive result as an acceptable risk [44]. This study was conducted from a healthcare perspective and consequently, did not include non-medical costs or indirect costs outside healthcare in our models. In addition to the harms of breast cancer screening, over-diagnosis which refers to a tumor detected on screening and leads to be over-treated, is still remaining concern. DCIS–a noninvasive form of breast cancer- is considered to be potential factor related to over-diagnosis. Due to the lack of information on DCIS in Vietnamese women and the fact that there is currently no method of confirmation about the probability as well as timing from DCIS to invasive cancer [45], we did not take DCIS into account in the models. As a consequence, the cost-effectiveness of mammography screening could be overestimated and considered carefully before the national mammography program is introduced. At the individual level, benefits and risks should be informed by health staffs and balanced information should be considered as part of the personal decision-making process.

Although this study has limitations, the findings still provide the first piece of evidence regarding the cost-effectiveness of mammography screening in Vietnamese women. Currently, national programs and medical services covered by health insurance are not based on health-economic assessment in Vietnam. However, the application of health-economic assessments to medical services is considered to be mandatory when choosing health services. The results, therefore, provide valuable information for decision-makers to choose cost-effective interventions for breast cancer while the Vietnamese healthcare budget is limited. However, before introducing national mammography screening, other factors should be taken into account to make the implementation in Vietnamese circumstances successful. The results revealed that the participation rate had a high impact on the effectiveness of national breast cancer screening. Hence, women’s values and preferences, such as the enhanced awareness of breast cancer, preferred setting, time, and cost of travelling to a screening unit, cultural promotion for screening, and changing beliefs of health service providers, should be part of the decision process to encourage the higher uptake rate [46, 47]. Other assumptions, such as 100% adequate treatment in patients, should be considered when cost-effectiveness results were interpreted in Vietnam. To reduce the mortality rate in breast cancer patients, the adequate treatment, along with mammography screening, should be noticed.

## Conclusions

Our analyses have illustrated that the first round of mammography screening for the 50–54 age group is the most cost-effective age-related screening scenario and furthermore, both screening for the 50–54 age group and the 55–59 age group are cost-effective with the Vietnamese ceiling threshold. For women aged 45–49 years, mammography screening is not cost-effective since the incidence rate of breast cancer and the sensitivity of mammography in women in this age group are lower than that of the other groups. The discount rate, mammography sensitivity, and transition probability from remission to distant recurrence in stage II have a large impact on the ICER in all four age groups. Further studies should be conducted to examine the effectiveness of mammography screening and provide epidemiological data on breast cancer in Vietnamese women.

## Supporting information

S1 Table Adjusted transition probabilities between health states in the Markov model. (DOCX)

S2 Table Cost-effectiveness of second round of mammography screening. (DOCX)
