# Supplementary material for: Cost-effectiveness of breast cancer screening using mammography in Vietnamese women
Source: PLoS One. 2018 Mar 26;13(3):e0194996. doi: 10.1371/journal.pone.0194996 (PMC5868837; doi:10.1371/journal.pone.0194996)
Supplement: S1 Table — (DOCX) [file pone.0194996.s001.docx]

**S1 Table. Adjusted transition probabilities between health states in the Markov model.**

| **Transition probabilities** | **Canada study** | **Adjusted** |
| --- | --- | --- |
| **45-49 group** |  |  |
| ***Stage I*** |  |  |
| *Remission to Local recurrence* | 0.01 | 0.013 |
| *Remission to Distant recurrence* | 0.000016 | 0.000021 |
| *Local recurrence to Distant recurrence* | 0.062 | 0.080 |
| *Local recurrence to breast cancer death* | 0.013 | 0.017 |
| *Distant recurrence to breast cancer death* | 0.555 | 0.719 |
| ***Stage II&III*** |  |  |
| *Remission to Local recurrence* | 0.018 | 0.023 |
| *Remission to Distant recurrence* | 0.024 | 0.031 |
| *Local recurrence to Distant recurrence* | 0.165 | 0.214 |
| *Distant recurrence to breast cancer death* | 0.386 | 0.500 |
| ***Stage IV*** |  |  |
| *Distant recurrence to breast cancer death* | 0.386 | 0.500 |
| **50-59 group** |  |  |
| ***Stage I*** |  |  |
| *Remission to Local recurrence* | 0.009 | 0.011 |
| *Remission to Distant recurrence* | 0.000025 | 0.0000296 |
| *Local recurrence to Distant recurrence* | 0.052 | 0.061 |
| *Distant recurrence to breast cancer death* | 0.137 | 0.162 |
| ***Stage II&III*** |  |  |
| *Remission to Local recurrence* | 0.016 | 0.019 |
| *Remission to Distant recurrence* | 0.105 | 0.124 |
| *Local recurrence to Distant recurrence* | 0.13 | 0.154 |
| *Distant recurrence to breast cancer death* | 0.423 | 0.500 |
| ***Stage IV*** |  |  |
| *Distant recurrence to breast cancer death* | 0.423 | 0.500 |

Adjusted transition probability was estimated using the equation:

**The 45-49 age group**

P_A_ = P_C_ x 0.5/ 0.386

Where:

P_A_: adjusted transition probability

P_C_: Transition probability from the Canadian study

0.5: survival probability of patients in stage IV

0.386: transition probability from distant recurrence to breast cancer death in stage IV (Canadian study)

**The 50-64 age group**

P_A_ = P_C_ x 0.5/ 0.423

Where:

P_A_: adjusted transition probability

P_C_: Transition probability from the Canadian study

0.5: survival probability of patients in stage IV

0.423: transition probability from distant recurrence to breast cancer death in stage IV (Canadian study)
