# Supplementary material for: Cost-effectiveness of breast cancer screening using mammography in Vietnamese women
Source: PLoS One. 2018 Mar 26;13(3):e0194996. doi: 10.1371/journal.pone.0194996 (PMC5868837; doi:10.1371/journal.pone.0194996)
Supplement: S2 Table — (DOCX) [file pone.0194996.s002.docx]

## **S2 Table. Cost-effectiveness of the second round of mammography screening.**

The second round was performed after two years of the first screening. The subsequent rate of the second screening and interval rate were assumed to be 0.48 times and 0.086 times the incidence rate of the first screening, respectively [1]. It was assumed that all women screened in the first round would participate in the second round of screening and that women with a false negative outcome breast cancer would be detected before the second round of screening. The interval cancer rate was assumed the same in the mammography screening group and the no screening group. Our assumptions in the first and second round of screening were similar to some of the assumptions made in previous studies [2, 3].

**S2 Table**. **Cost and effect of screening policies in the second round.**

| **Strategy** | **Cost (US$)** | **Life year gained** | **ICER (US$)** | **INMB (US$)** |
| --- | --- | --- | --- | --- |
| **Women aged 45-49 years** |  |  |  |  |
| No screening | 181,008 | 100,655 |  |  |
| Mammography screening | 2,282,332 | 100,814 | 13,234.78 | -1,095,855 |
| **Women aged 50-54 years** |  |  |  |  |
| No screening | 162,018 | 99,893 |  |  |
| Mammography screening | 2,253,809 | 100,304 | 5,090.83 | 510,357 |
| **Women aged 55-59 years** |  |  |  |  |
| No screening | 154,465 | 99,104 |  |  |
| Mammography screening | 2,234,429 | 99,416 | 6,656.81 | -101,207 |
| **Women aged 60-64 years** |  |  |  |  |
| No screening | 123,333 | 97,597 |  |  |
| Mammography screening | 2,165,115 | 97,825 | 8,923.69 | -592,785 |

**References**

1. Sripaiboonkij N, T.B., Promthet S, Kannawat C, Tangcharoensathien V, Ansusing T, et al., *Breast Cancer Detection Rate, Incidence, Prevalence and Interval Cancer-related Mammography Screening Times among Thai Women.* Asian Pac J Cancer Prev, 2016. **17**(8): p. 4137-41.

2. Gocgun Y, B.D., Taghipour S, Montgomery N, Harvey BJ, Jardine AK, Miller AB., *Cost-effectiveness of breast cancer screening policies using simulation.* The breast, 2015. **24**(4): p. 440-448.

3. Shahpar Haghighat, M.E.A., ParvinYavari, MehdiJavanbakht, ShahramGhaﬀari, *Cost-effectiveness of three rounds of mammography breast cancer screening in Iranian women.* Iran J Cancer Prev, 2016. **9**(1).
